# Integrative transcriptomic and physiological assessment of nanoencapsulated carvacrol and thymol oil as an antioxidant in thermal-stressed white shrimp (*Litopenaeus vannamei*)

**DOI:** 10.1007/s11259-025-11042-x

**Published:** 2026-01-22

**Authors:** Adrián Ríos-Ortiz, María F. Barragán-Longoria, Erika Magallón-Gayón, Rocio A. Chavez-Santoscoy, Andrea Manriquez-Patiño, Rafael Vazquez-Duhalt, María Teresa Viana

**Affiliations:** 1 Doctorado en Medio Ambiente y Desarrollo, Instituto de Investigaciones Oceanológicas (IIO), Universidad Autónoma de Baja California (UABC), Km 107 carretera Tij/Eda, Ensenada, 22860 Baja California México; 2 Tecnológico de Monterrey, Escuela de Ingeniería y Ciencias, Ave. Eugenio Garza Sada 2501 Sur, Col: Tecnológico, 64700 Monterrey, N.L México; 3 Doctorado en Oceanografía Costera, Facultad de Ciencias Marinas, (UABC), Ensenada, México; 4 Centro de Nanociencias y Nanotecnología, Universidad Nacional Autónoma de México, Ensenada, BC México; 5 IIO, UABC, Ensenada, México

**Keywords:** Shrimp, Transcriptomic, Glutathione peroxidase, Catalase, Stress

## Abstract

The present study aimed to assess the effects of chitosan nanocapsules loaded with synthetic carvacrol and thymol (structurally analogous to those in oregano oil) on the antioxidant and molecular response of *Litopenaeus vannamei* subjected to acute thermal stress. Chitosan nanocapsules (of 246 nm) were prepared and incorporated into shrimp feed at eight concentrations in triplicate: 0.0 (0ChNP), 0.033mg g^− 1^ (0.03ChNP), 0.1mg g^− 1^ (0.1ChNP), 0.2mg g^− 1^ (0.2ChNP), 0.3mg g^− 1^ (0.3ChNP), 0.467mg g^− 1^ (0.5ChNP), 0.633mg g^− 1^ (0.6ChNP), and 0.8mg g^− 1^ (0.8ChNP). Twenty-four aquariums, each containing three shrimp (10.25 ± 1.14 g), were fed for two weeks. The first 8 days at 27 ± 0.5 °C, followed by a gradual temperature increase to 39 °C. Thereafter, shrimps were sampled, and the hepatopancreas dissected and divided into two, and individually stored in RNAlater for transcriptomic and qPCR analysis. The groups 0ChNP, 0.03ChNP, and 0.3ChNP were selected for transcriptomic analysis based on their physical appearance, and mortality rate. At 0.03ChNP, significant upregulation of genes associated with innate immunity, vesicular trafficking, and autophagy was detected, suggesting an activated protective response. On the other hand, the qPCR on anti-oxidative enzymes, catalase and glutathione peroxidase, showed lower levels than in the control experiments suggesting reduced oxidative stress when chitosan nanocapsules were supplied, especially with 0.3ChNP. In conclusion, chitosan nanocapsules delivering carvacrol and thymol at 0.3 mg g^− 1^ of feed modulate distinct molecular pathways that contribute to thermal stress resilience in shrimp. Doses below or above this threshold were ineffective or detrimental under the conditions tested.

**Supplementary Information:**

The online version contains supplementary material available at 10.1007/s11259-025-11042-x.

## Introduction

In aquaculture, worldwide, the white shrimp (*Litopenaeus vannamei*) is a fundamental species due to its high demand and nutritional properties (Li et al. 2021). However, the intensification of its culture can generate negative scenarios, such as the accumulation of waste, including nutrients and organic compounds such as NH_3_ and NO_2_, resulting in anoxia and a toxic environment, affecting its production (Henriksson et al. 2018; Obirikorang et al. 2024). Likewise, climate change has been associated with temperature increase in production sites exacerbating all metabolic processes (Islam et al. 2019).

Stress conditions can generate a loss of balance between reactive oxygen species (ROS) and the antioxidant capacity of the shrimp (Li et al. 2016). This prolonged imbalance can permanently damage the genetic material (Li et al. 2016). ROS are highly reactive molecules that are generally produced in mitochondria during the process of cellular respiration and have a role as signalers (Snezhkina et al. 2019); however, they are also produced in response to stressors such as heat stress, ultraviolet radiation, and chemical pollutants (Halliwell 2020). Some studies have shown that supplementing antioxidants can mitigate these effects by reducing ROS accumulation and consequently, oxidative damage (Wu et al. 2023; Dokou et al. 2023). Antioxidants not only neutralize the ROS produced in the organisms but can also protect the feed from any other oxidant produced by rancidity of the ingredients during storage. Carvacrol and thymol, are one of these antioxidants normally found in oregano oil, compounds that have been widely applied in aquaculture to improve growth performance, enhance innate immunity responses, survival, and resistance to environmental stress (Novriadi et al. 2023). Previous studies have shown that oregano oil improves antioxidant and immune responses in fishes like *Oreochromis niloticus* (Magouz et al. 2022), *Oncorhynchus mykiss* (Dokou et al. 2023), *Salmo labrax* (Özel et al. 2024) and *Cyprinus carpio* (Zhang et al. 2020), as well in crustaceans like *Procambarus clarkii* (Guo et al. 2025) and *Macrobrachium rosenbergii* (Ballester et al. 2025), helping to reduce oxidative stress and improving parameters such as growth and survival, but there is a lack of information in white shrimp.

Despite its broad and potential use, several commercial herbal products with antioxidant properties for aquaculture lack clear ingredient information and are usually loaded with a high content of excipients to easily blend in feed mixtures. Further, since the bioactive compounds are blended in an adsorbent surface, the antioxidant properties would probably be neutralized in the feed during preparation (Olmedo et al. 2015), storage (Turek and Stintzing 2012) or even along the digestive system (Lima et al. 2019), leading to a reduced protective role in stress events.

Nanoencapsulation technologies offer a promising solution by protecting bioactive ingredients and improving the stability and bioavailability of these compounds, enabling their release and increasing effectiveness (Dokou et al. 2023; Wu et al. 2023). Currently, the effects of nonencapsulated oregano oil on gene expression related to thermal and oxidative stress in *L. vannamei* is being studied (Magouz et al. 2022; Wu et al. 2023), but the molecular mechanisms, especially those involving heat shock proteins (HSPs) and antioxidant responses, remain poorly understood (Wu et al. 2023). Within this context, omics strategies, especially transcriptomic approaches, such as RNA sequencing (RNA-seq) and quantitative PCR (qPCR), are powerful tools to characterize molecular responses in aquatic organisms exposed to environmental stressors (Chandhini and Rejish-Kumar 2018). This approach allows us to understand gene expression patterns involved in different metabolic pathways, and their modulation by dietary interventions such as nanoencapsulated antioxidant compounds.

Therefore, the present study aims to fully understand how carvacrol and thymol, protected by nanoencapsulation in the feed, regulate antioxidant responses under heat shock as thermal stress in *L. vannamei*, at a transcriptomic and molecular level. This contributes to the development of innovative and sustainable strategies to mitigate heat stress in aquaculture.

## Materials and methods

### Experimental conditions

Shrimp handling was carried out in accordance with the ethical guidelines established by the Committee on Ethics in the Use of Animals of the Autonomous University of Baja California, under the supervision of the corresponding institutional committee. The organisms were provided by a commercial hatchery (AquaPacific S.A. de C.V., Mazatlán, Mexico).

Before starting the experiment, the shrimp were kept in a recirculation system for acclimatization for seven days. Twenty-four aquaria, 20-L each were defined as the experimental unit (EU). All connected into a RAS system with additional aeration, a biofilter, and a heater to maintain a controlled temperature (minimum 27 ± 0.5 °C), containing a UV light to improve water quality. The parameters such as dissolved oxygen (7.0 ± 0.14 mg L^− 1^), and salinity (35 ± 0.2 PSU) were daily monitored to ensure the conditions of the experiment, using a YSI-55 optical multiparameter system (YSI Inc., Yellow Springs, OH, USA). Additionally, ammonia, nitrite, and nitrate concentrations were measured three times per week using API test kits (Mars Fishcare Inc., Chalfont, PA, USA), ensuring that they remained close to zero. The pH level was checked weekly with a Thermo Scientific Orion 4-Star meter (Thermo Scientific, Waltham, MA, USA).

The temperature regime was designed to mimic diurnal fluctuations observed in commercial shrimp farms in Northwest Mexico during the summer months, where temperatures can exceed 35 °C during the day and drop at night (Ruacho et al. 2012). The highest reached temperature was at 39 °C included at last day was chosen to simulate extreme heat event linked to climate change, thereby testing the resilience provided by the dietary supplements under realistic stress conditions. During the first week shrimp were kept at a constant temperature (27 ± 0.5 °C). From the second week, (day 8 to day 10), the water temperature was raised to 31.4 ± 1.5 °C during the day for 4 h, decreasing to 27.4 ± 0.9 °C at night. From day 11 to day 13, water reached a maximum temperature of 36.2 ± 2.0 °C decreasing to 27.5 ± 0.03 °C at night. On day 14, a final maximum temperature challenge was carried out, reaching 39 °C for 1:30 h and decreasing to 38 °C for 4:30 h. After this final challenge, all shrimp were individually sampled per EU without pooling.

Each hepatopancreas was cut in half, and each half was stored in RNAlater separately and identified. Survival was assessed by averaging the survival obtained from each aquarium, which was monitored daily and reported as percentage.

$$Survival\;(Sv,\;\%):\;Sv=\;(Nf\times100)\;/\;Ni$$

Where Nf is the number of shrimps at the end of the study, and Ni is the initial number of organisms.

Furthermore, the physical aspect of the hepatopancreas was monitored at the end of the trial, specifically the redness aspect of the organ.

### Synthesis of Chitosan nanoparticles loaded with carvacrol and thymol

The synthesis of chitosan nanoparticles with carvacrol and thymol followed the protocol proposed by Quester et al. (2022). Pure chitosan (2.5 mg mL^− 1^ Sigma-Aldrich, St. Louis, MO, USA) was dissolved in an acetic acid solution at 2%, and stirred for 24 h at room temperature, then filtered to remove impurities. A commercial synthetic formulation of oregano oil (Ghaziabad Aromatics, India).

To synthesize the nanoparticles, the synthetic oregano oil was diluted in polyoxyethylene (20) sorbitan monooleate and water in a 1:1:2 ratio, respectively, under stirring to form an emulsion for one hour. This solution (200 mL) is then added to 10 mL of chitosan solution and homogenized. Then, 1 mL (0.25 mg mL^− 1^) of tripolyphosphate (TPP) was added to initiate ionic cross-linking with chitosan, forming the nanoparticle structure. Stirring was allowed for 1 h. Finally, 100 µL of 2.5% glutaraldehyde was added to crosslink the nanoparticles by covalent bonds, and then the excess glutaraldehyde was neutralized by lysine to improve their biocompatibility (Gallardo et al. 2019). Finally, the nanocapsules were centrifuged, and the pellet containing the nanoparticles was resuspended in a phosphate buffer at pH 6 for later use.

The commercial synthetic oregano oil (Ghaziabad Aromatics, India) was quantified alone, and extracted from the nanocapsules, using gas chromatography, resulting in a concentration of 22.2% of bioactive molecules from the total pure oil provided, corresponding to 36.3% carvacrol and 36.3% thymol.

### Nanoparticles characterization

Hydrodynamic diameter, zeta potential, and polydispersity were determined by Dynamic Light Scattering (DLS) on a Zetasizer nano ZS90 instrument (Malvern, UK).

Carvacrol and thymol content were determined after acid hydrolysis of nanocapsules by gas chromatography (Agilent 7820 A Gas Chromatograph) equipped with a flame ionization detector (FID). The samples were analyzed through a Zebron Inferno 20 m x 0.18 mm x 0.18 μm column (Phenomenex, Los Angeles, CA, USA). The GC program started at 50 °C (2 min), followed by a temperature ramp of 10 °C/min to 375 °C (5 min). The splitless injector was set up at 360 °C and the detector at 400 °C. Helium was used as a carrier gas at a constant flow of 0.9 mL min^− 1^.

On the other hand, the concentration of chitosan in the nanoparticles was carried out by UV-Vis spectrophotometry (Lambda 25, Perkin Elmer, Waltham, MA, USA), based on the method proposed by González-Davis et al. (2023).

The antioxidant activity was estimated according to Chanput et al. (2016). This method is based on the inhibition of the oxidation of ABTS (2,2′-azino-bis(3-ethylbenzothiazoline-6-sulfonic acid) mediated by methemoglobin and hydrogen peroxide. The inhibition extent of ABTS radical cation is expressed in Trolox equivalent units and butylhydroxytoluene (BHT) equivalents.

### Diets formulation and experimental design

Once the nanocapsules were synthesized and characterized, eight isoprotein and isolipid moist diets were made. Since the trial was designed for a short period without considering growth or any overall performance, the diets were made with fishmeal and gelatin to avoid any interaction with other ingredients. However, considering that *L. vannamei* is a carnivore species, the content of protein was based on NRC (2011) guidelines (40% crude protein and 7% crude lipids, on a wet weight basis, ensuring that all organisms were perfectly fed under similar circumstances. The experimental groups were divided into eight diets with different nanoparticles concentrations given as mg per g feed to become the treatments (Table 1), 0.0 (0ChNP), 0.033 mg g^− 1^ (0.03ChNP), 0.1 mg g^− 1^ (0.1ChNP), 0.2 mg g^− 1^ (0.2ChNP), 0.3 mg g^− 1^ (0.3ChNP), 0.467 mg g^− 1^ (0.5ChNP), 0.633 mg g^− 1^ (0.6ChNP), and 0.8 mg g^− 1^ (0.8ChNP). The diets were manufactured at the LINDEAACUA IIO-UABC facilities (Ensenada, Mexico) and maintained at −20 °C until used. Three shrimp (10.25 ± 1.14 g) were stocked in each of the 24 aquariums. The number of shrimps per EU was determined according to their size and available volume, surface area to avoid density stress and ensure adequate oxygen availability (D’Abramo et al. 2000). Each treatment had three replicates, adequately for eight treatments and samples were handled individually. The feeding rations were adjusted according to its feed ingestion. Shrimp were fed by hand four times a day to satiation to ensure consumption, and excess feed was removed before offering the next ration.

**Table 1:** Ingredient and proximate composition of dietary treatments

Ingredients (g kg^− 1^ DM)	0ChNP	0.03ChNP		0.1ChNP		0.2ChNP		0.3ChNP		0.5ChNP		0.6ChNP		0.8ChNP	
Fishmeal	80	80		80		80		80		80		80		80	
Gelatin^b^	10	10		10		10		10		10		10		10	
Cornstarch^c^	5	5		5		5		5		5		5		5	
Fish oil	5	5		5		5		5		5		5		5	
ChNP (mg g^− 1^)	0	0.033		0.1		0.2		0.3		0.47		0.63		0.8	
Total	100	100		100		100		100		100		100		100	
***Proximal Composition as fed (g kg*** ^***− 1***^ ***)***															
Crude protein	40														
Crude lipids	7.03														
Ash	5.20														
Moisture	55														

*Treatments: 0ChNP (0 mg g^− 1^), 0.03ChNP (0.033 mg g^− 1^), 0.1ChNP (0.1 mg g^− 1^), 0.2ChNP (0.2 mg g^− 1^), 0.3ChNP (0.3 mg g^− 1^), 0.5ChNP (0.467 mg g^− 1^), 0.6ChNP (0.633 mg g^− 1^) y 0.8ChNP (0.8 mg g^− 1^); ^b^Progel Mexicana S.A. de C.V., León, Guanajuato, Mexico; ^c^Ingredión Mexico S.A. de C.V

### RNA sequencing

#### RNA extraction

Thirty mg of shrimp hepatopancreas was individually weighed and homogenized in Fastprep-24 homogenizer (MP Biomedicals). RNA was isolated using the RNeasy Plus Mini Kit (Qiagen) following the supplier’s instructions. Concentration was measured with Qubit RNA HS Assay Kit (Invitrogen), and purity was assessed with a NanoDrop1000 (Thermo Fisher Scientific). To assess RNA integrity, all samples were analyzed with an R1 cartridge in a Qsep400 (BiOptic Inc).

#### RNA library Preparation and sequencing

Transcriptome libraries were prepared with the TruSeq Stranded Total RNA Library Prep with Ribo-Zero Gold (Illumina) and subsequently quantified with the Qubit dsDNA HS Assay Kit (Invitrogen). Library size was analyzed on a QSep 400 (BiOptic), and sequencing was performed on a NovaSeq 6000 in a 2 × 100 PE configuration.

#### Transcriptome assembly and functional annotation

To determine sequencing quality, raw sequences were evaluated using FastQC (Babraham Bioinformatics). De Novo assembly of the *L. vannamei* transcriptome was performed using the reads from all the pooled samples. Trinity v2.13.2 (Haas et al. 2013) was used with default parameters (Grabherr et al. 2011). The assembled transcriptome was evaluated to remove chimeras and duplicates with CD-Hit (Fu et al. 2012). Then, the CD-HIT output file was run on TransDecoder 5.7.0 (Haas et al. 2013), using the default settings, to identify long open read frames (ORFs) in assembled transcripts. The functional annotation was carried out with Blast, comparing against the Penaeus database in Uniprot. The quality and completeness of the transcriptome were evaluated with version the BUSCO software v.5.5.0 (Manni et al. 2021). This analysis was run in transcriptome mode, using the arthropoda_odb10 lineage dataset and with default settings.

#### Differential gene expression analysis and enrichment analysis

Differential gene expression between each experimental group and the control was performed with DESeq2 (Love et al. 2014), using the assembled transcriptome as reference. DEGs were identified using a threshold of false discovery rate (FDR) < 0.05 and a fold-change > 1.

Functional enrichment analysis of DEGs was conducted using the ShinyGO v0.82 platform (Ge et al. 2020). Enriched pathways were identified by mapping the GO Biological Process, GO Molecular Function and GO Cellular Component for the *P. vannamei* database. Default parameters were used, including a false discovery rate (FDR) threshold of < 0.05 to determine statistical significance.

### Quantitative PCR

From each sample, RNA was individually extracted from the hepatopancreatic tissue samples, previously preserved in RNAlater (Ambion), using the PureLink^®^ RNAlink Minikit (Ambion). Genomic DNA (gDNA) was removed using PureLink^®^ DNase (Invitrogen) following the manufacturer’s instructions. The tissue was homogenized using a micropistil before extraction. The spectrophotometer assessed RNA purity and concentration (Nanodrop^®^ LITE, Thermo Fisher Scientific INC., Wilmington, USA). Only RNA samples with OD_260nm_/OD_280nm_ ratios between 1.9 and 2.1 were used for expression quantification.

The primers used for the different genes were selected from López et al. (2023) based on the mRNA sequences available in the GenBank database of the National Center for Biotechnology Information (NCBI; see Table 2). In addition to the target genes, three potential reference genes were included: ribosomal protein L8 (rpL8), elongation factor 1-alpha (Ef1α), and glyceraldehyde-3-phosphate dehydrogenase (GAPDH).

**Table 2:** Genes tested through qPCR in hepatopancreas tissue of *Litopenaeus vannamei* within the different dietary treatments containing Chitosan nanoparticles with oregano extract

Gene	GenBank	Forward Sequence (5’−3’)	Reverse Sequence (5’−3’)	Size (pb)	e
***Glyceraldehyde-3-phosphate dehydrogenase: LvGAPDH***	MG787341.1	TCGGCAAGGAGTGCTCTTAT	GCCTTAGCGTCAAAGATGGA	150	2.01
***Elongation factor 1*** ***alpha: LvEF1α***	GU136229	GAAATCCGACAACATGGGCT	CCAATCTTGTACACGTCCTG	162	1.99
***Ribosoma protein L8***: ***LvL8***	DQ316258	ATGAACCCTGTAGAGCATCCTC	CTTTGTACCACGGATGAGACCA	141	2.15
***Manganese superoxide dismutase: LVcMnSOD***	DQ029053	CGTAGAGGGTATTGTCGTACAG	AGGGCGTTGAAATCATACTTGAG	152	1.97
***Catalase: LVCat***	AY518322	GCGACCAGAAACAACACACC	CTTGATGCCTTGGTCCGTCT	165	1.92
***Glutathione peroxidase: LVGSH-Px***	AY973252.2	GGACTTCCACCAGATGAACC	CTCGAAGTTGTTCCCAGGAC	153	2.02

The analysis proceeded as follows: 500 ng of total RNA was reverse transcribed in a 20 µL reaction using the High-Capacity cDNA Reverse Transcription Kit (Applied Biosystems; Carlsbad, CA, USA) in a 48-well Verity thermal cycler (Applied Biosystems). The reverse transcription program consisted of 10 min at 25 °C, 120 min at 37 °C, 5 min at 85 °C, followed by a holding period at 4 °C. After conducting stability analyses using gNorm software, rpL8 was chosen as the housekeeping gene. The efficiency values for each gene’s standard curve were calculated with the formula $\:E={(10}^{-1/slope})$−1 from serial dilutions (1:10) of cDNA.

Quantitative PCR (qPCR) reactions were performed using 1 ng of cDNA, sense and antisense primers (200 nM each), and SYBR^®^ Select Master Mix (Applied Biosystems). These reactions were carried out at a volume of 10 µL using 48-well MicroAmp^®^ Fast Optical Reaction Plates (Applied Biosystems), which were covered with MicroAmp^®^ Optical Adhesive Film (Applied Biosystems). Relative gene quantification was calculated using a modified algorithm based on the classical ΔΔCt method, considering the amplification efficiency for each target and reference gene. This was done using an automated threshold and an adjusted baseline to determine Ct values.

The PCR conditions included an initial denaturation and polymerase activation step at 95 °C for 10 min, followed by 40 cycles of denaturation for 15 s at 95 °C, and annealing and extension for 45 s at 60 °C. A final denaturation curve was performed from 60 °C to 95 °C for 20 min to check for primer-dimer artifacts.

### Statistical analysis

The normality and homoscedasticity of each parameter were evaluated. Parametric data were compared among the different nanocapsule concentration treatments using a one-way analysis of variance (ANOVA) followed by Tukey’s significant difference (Tukey’s HSD) test (IBM SPSS Statistics V23.0.0 Copyright IBM Corporation 1989, 2011, USA). Data normality and homoscedasticity were confirmed using Shapiro-Wilk and Levene’s tests, respectively, thus justifying the use of parametric tests such as ANOVA. Then a polynomial (quadratic) regression was performed among treatments, applying the significance value of *P* < 0.05 for all statistical tests. For the survival rate, the average values were arcsine-transformed. Since normality was not met, a Kruskal-Wallis test was applied (IBM SPSS Statistics V23.0.0, Copyright IBM Corporation 1989, 2011, USA).

## Results

### Nanoparticles characterization

**Fig. 1:**
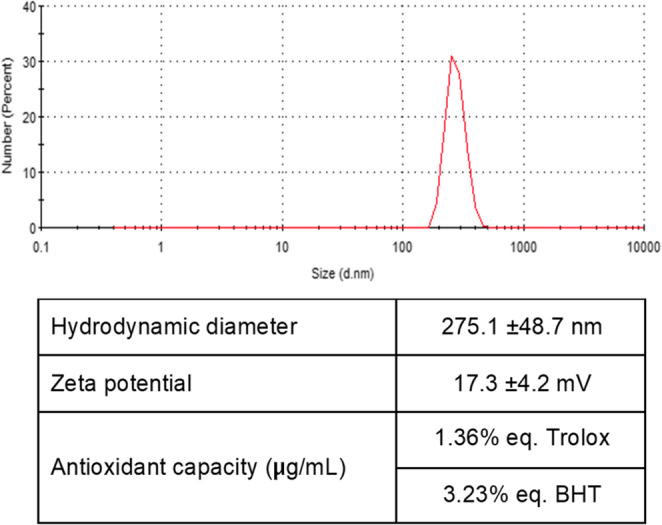
Properties of the chitosan nanoparticles loaded with carvacrol and thymol

After the synthesis of nanocapsules containing carvacrol and thymol, the size distribution measured by DLS showed an average hydrodynamic diameter of 275.1 nm (Fig. 1). The zeta potential, which represents the charge density on the particle surface, indicates that the nanoparticle suspension is moderately stable. In addition, it is evident that the preparation has an antioxidant capacity (Fig. 1).

### Physiological response and survival

During the last six days of experimentation, when temperature was raised on first three days (day 8 to 10) up to 31.4 °C, no shrimps died, whereas the last three days raising the temperature for four hours up to 39 °C eight shrimps out of 72 died from the different experimental treatments, without showing a significant difference.

Nevertheless, during sampling, it was observed that some hepatopancreases displayed reddish coloration in some treatments. In frequency, reddish hepatopancreases were found as follows: 3 in 0.8ChNP, 1 in 0.6ChNP, 2 in 0.5ChNP, 1 in 0.2ChNP, 1 in 0.1ChNP, and 1 in 0.03ChNP (Table 3). Therefore, two experimental groups were chosen to be tested against the Control group for the transcriptomic analysis. The treatment where no red coloration in the hepatopancreas was chosen to be compared with the Control group, and the lowest doses being the 0.03ChNP vs. Control comparison revealing 13 up-regulated genes where eight are important (Table 4). Whereas the 0.3ChNP vs. Control comparison resulted in 28 up-regulated and 29 down-regulated genes.

**Table 3:** Survival rate and physical hepatopancreas condition of the hepatopancreas at the end of the experimental procedure

Treatments	Survival rate (%)	Physical appearance of hepatopancreas (%) (redness)
0ChNP	77.78	0
0.03ChNP	77.78	25
0.1ChNP	88.89	16.6
0.2ChNP	88.89	16.6
0.3ChNP	100.00	0
0.5ChNP	88.89	16.6
0.6ChNP	100.00	16.6
0.8ChNP	88.89	16.6
*P value*	0.467	

**Table 4:** Concurrent genes encountered through transcriptomic analysis in *Litopenaeus vannamei* among the different dietary treatments after being fed with different amounts of Chitosan nanoparticles with oregano extract

Gene	Localization	Main Function	Associated Process	Treatments
TRS20 (TRAPP Subunit 20)	Nucleus (cytoplasmic protein)	Vesicular trafficking (ER → Golgi)	intracellular transport	0.03ChNP, 0.3ChNP, 0.8ChNP
mt-Co1 (Cytochrome c Oxidase I)	Mitochondria	Energy production (oxidative phosphorylation)	Cellular respiration	0.03ChNP, 0.3ChNP
Transferrin-like	Plasma, cell membrane	Iron transport	Iron homeostasis	0.3ChNP, 0.8ChNP
C-type lectin-1	Cell membrane	Pathogen recognition	Innate immune response	0.03ChNP, 0.3ChNP
Arylsulfatase B y D-like	Lysosomes	Degradation of sulfates into macromolecules	Glycosaminoglycan catabolism	0.03ChNP, 0.3ChNP
Glutathione S-transferase T2-like	Cytoplasm	Cellular detoxification by conjugation with glutathione	Response to oxidative stress	0.3ChNP
Histone-lysine N-methyltransferase Suv4-20-like	Core	Histone modification (lysine methylation)	Regulation of gene expression	0.3ChNP
Sodium/glucose cotransporter 5-like (SGLT5-like)	Cell membrane (kidney, intestine)	Glucose and sodium transport	Renal reabsorption of glucose	0.3ChNP

**Fig. 2:**
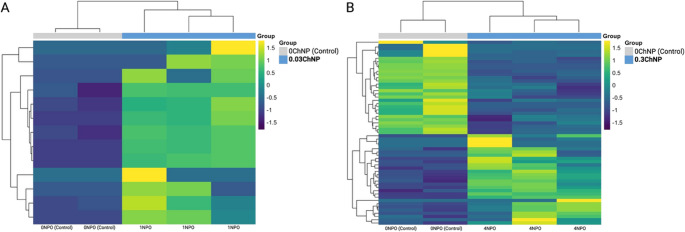
Heatmaps of differentially expressed genes in L. vannamei exposed to oregano oil nanoparticles. (**A**) Comparison between control (0ChNP) and treatment 0.03ChNP (0.033 mg g−1). (**B**) Comparison between control (0ChNP) and treatment 0.3ChNP (0.3 mg g−1). Each column represents an individual sample

For the de novo transcriptome assembly results distinct expression profiles were observed between the control group 0ChNP and the ChNP-treated groups (Fig. 2). In the 0.03ChNP group (0.033 mg g^− 1^), samples clustered separately from controls, exhibiting a distinct gene expression pattern. In contrast, the 0.3ChNP group (0.3 mg g^− 1^), defined as the optimal dose revealed a more balanced expression pattern with fewer genes showing strong changes in expression.

**Fig. 3:**
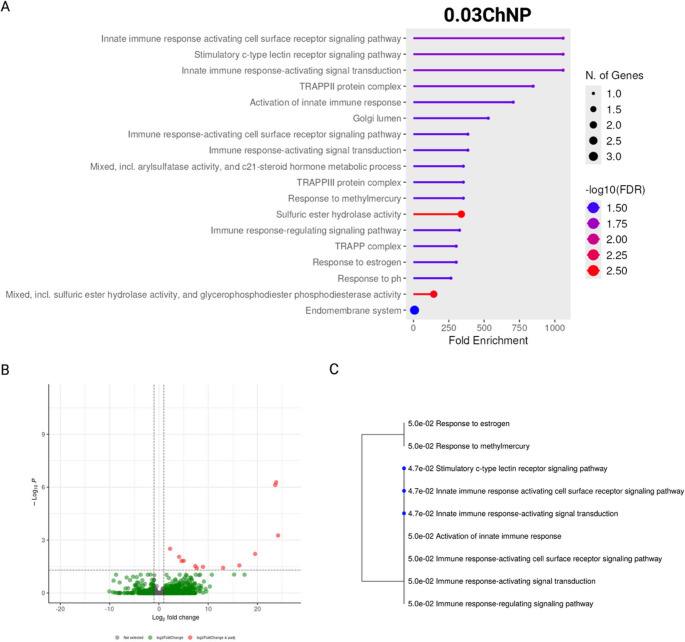
Enrichment analysis of differentially expressed genes in *Litopenaeus vannamei* exposed to oregano oil nanoparticles 0.03ChNP (0.033 mg g−1) under thermal stress, compared to control (0ChNP). (**A**) Enriched gene sets (all categories). (**B**) Volcano plot for differentially expressed genes (DEGs). (**C**) Tree plot showing enriched Biological Process (GO) terms

The enrichment analyses performed using ShinyGO v0.82 for both treatment groups (0.03ChNP and 0.3ChNP) compared to the control (0ChNP) resulted in an enrichment in the 0.03ChNP group, with a strong overrepresentation of gene ontology (GO) terms such as cell surface receptor signaling pathways and C-type lectin receptor signaling. Additionally, the complexes TRAPPII and TRAPPIII were also enriched (Fig. 3A, B and C).

**Fig. 4:**
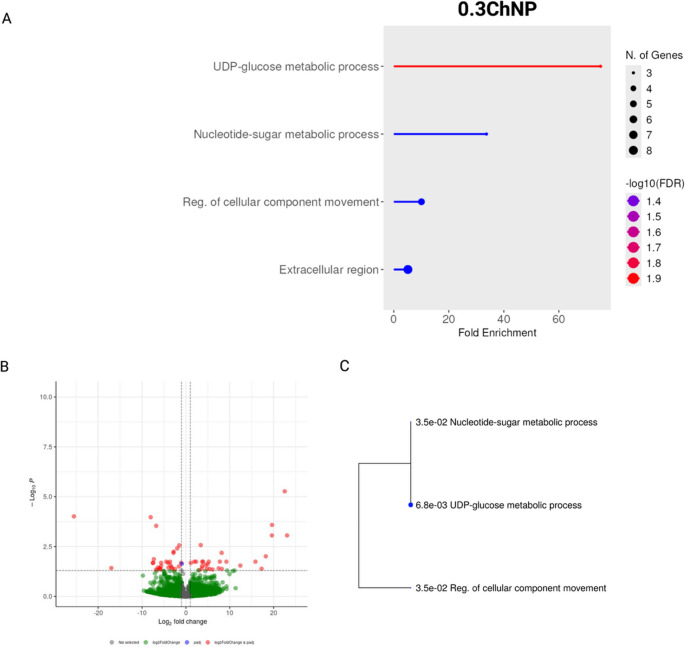
Enrichment analysis of differentially expressed genes in *Litopenaeus vannamei* exposed to oregano oil nanoparticles 0.3ChNP (0.3 mg g−1) under thermal stress, compared to control (0ChNP). (**A**) Enriched gene sets (all categories). (**B**) Volcano plot for differentially expressed genes (DEGs). (**C**) Tree plot showing enriched Biological Process (GO) terms

In contrast, the 0.3ChNP group, previously defined as the optimal dose based on physiological observations, exhibited a different enrichment profile. For instance, the GO terms, including UDP-glucose and nucleotide-sugar metabolic processes, as well as the regulation of intracellular transport and the endoplasmic reticulum unfolded protein response (UPR) were upregulated (Fig. 4A, B and C).

**qPCR validation**.

To validate the RNA-seq results, quantitative PCR (qPCR) was performed on a subset of genes. Selected targets included genes involved in oxidative stress and antioxidant defense, such as manganese superoxide dismutase (Mn-SOD), catalase (CAT), and glutathione peroxidase (GSH-Px).

The expression of catalase (CAT), and glutathione peroxidase (GSH-Px) at different treatment doses (0ChNP, 0.03ChNP, 0.3ChNP, and 0.8ChNP), resulted in a polynomial model response. In the case of mnSOD, despite being expressed in all treatments, it failed to show a polynomial trend (*R* = 0.15), and therefore it was not reported.

**Fig. 5:**
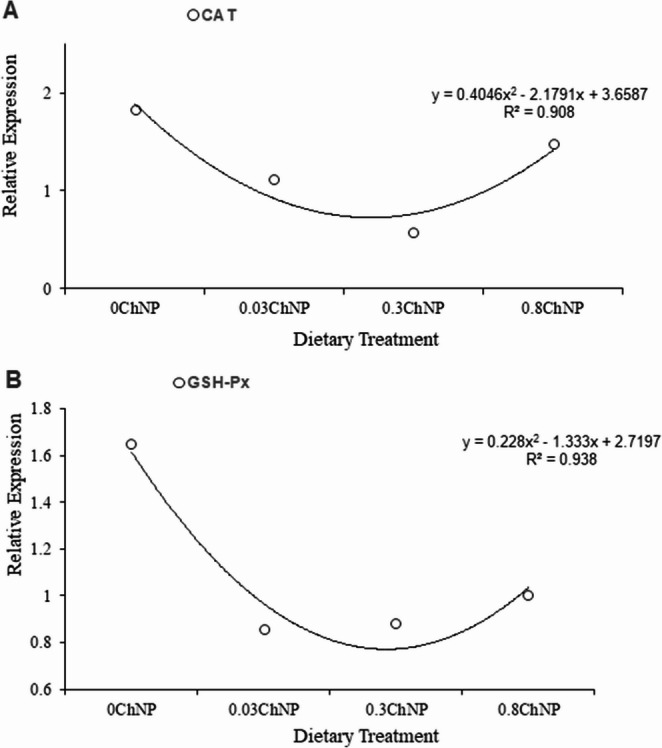
Gene expression of CAT (**A**) and GSH-Px (**B**) after qPCR from the hepatopancreas tissue of *Litopenaeus vannamei*, after being fed with the dietary treatments including the control group with different concentrations of chitosan nanoparticles with oregano extract

For CAT (Fig. 5A), expression showed an inverted second-order polynomial curve, with a maximum value at 0ChNP, a progressive decline to a minimum at 0.3ChNP, and a partial recovery at 0.8ChNP. Finally, GSH-Px (Fig. 5B) showed a similar, albeit less pronounced, trend. Expression decreased markedly from 0ChNP to 0.03ChNP, remaining low at 0.3ChNP, and with a slight recovery at 0.8ChNP.

## Discussion

The findings of this study reveal that dietary supplementation with chitosan nanoparticles (ChNP) encapsulating carvacrol and thymol induces a multi-level response in *Litopenaeus vannamei* under thermal stress. This includes dose-dependent modulation of antioxidant-related genes, transcriptomic reprogramming, and observable physiological changes, particularly hepatopancreatic pigmentation.

The 0.3ChNP (0.3 mg g^− 1^ feed) treatment was identified as the optimal concentration under our experimental conditions since yielded a 100% survival and no hepatopancreatic coloration, commonly associated with oxidative damage or injury by an infection (Zhang et al. 2023). Whereas, both lower and higher doses led to appearance of reddish hepatopancreas, indicating a U-shaped response curve, suggesting a hormesis effect (Ritchie and Friesen 2022). At low doses (0.03ChNP), the antioxidant stimulus was insufficient to counteract thermal stress, leading to oxidative damage and immune overactivation. Whereas at high doses (≥ 0.5ChNP), carvacrol and thymol may exhibit a pro-oxidant outcome, exacerbating the cellular stress (Qian et al. 2022). Only at 0.3 mg/g of nanoparticles inclusion provided an optimal redox balance, emphasizing the importance of precise dosing in the present study (Table 3).

The thermal challenge was designed to progressively simulate the temperature fluctuations commonly encountered in commercial shrimp farms due to climate change. These temperature profiles are consistent with recorded fluctuations in shrimp farms in arid regions such as Northwest Mexico, where daytime temperatures can exceed 35 °C and drop significantly at night (Yuniartik et al. 2022; Muralidhar et al. 2021). In the present work, the thermal heat stress was performed during 6 days from 31 to 36 °C with a phase of acute exposure at 39 °C during the previous day of sampling.

Physiologically, hepatopancreatic pigmentation served as a visible biomarker of oxidative stress. Reddish coloration is often associated with cellular damage, according to Abraham et al. (2013) and Zhang et al. (2023). The hormesis response obtained here is characteristic of phenolic compounds (Snezhkina et al. 2019), suggesting that doses deviating from the optimal concentration compromise cellular homeostasis, potentially due to overactivation of stress pathways or prooxidant effects at higher levels. Furthermore, since no mortality was observed in the 0.3ChNP group in our study and no reddish color in the hepatopancreas was found. Thus, we believe that this dose plays a protective role due to its strengthening of antioxidant pathways and supports its designation as the “optimal dose”.

The transcriptomic analysis focused on the three representative groups: control (0ChNP), low-dose (0.03ChNP), and optimal-dose (0.3ChNP), where chosen to reduce complexity and highlight biologically relevant contrasts. Differential expression analysis revealed distinct transcriptional profiles among treatments. The transcriptomic contrasts among treatments revealed that nanoencapsulated carvacrol and thymol elicited dose-dependent molecular signatures in thermally stressed *L. vannamei*. At the low dose (0.03ChNP), shrimp showed broad transcriptional activation of genes involved in innate immunity (e.g., pattern recognition receptors, antimicrobial peptides), vesicular trafficking, and autophagy, consistent with a compensatory protective response. This pattern aligns with the distinct clustering observed in the PCA and hierarchical heatmap, where 0.03ChNP samples were separated from both the control and optimal-dose groups, suggesting a heightened, yet potentially stressful, transcriptional state. In both cases, hierarchical clustering segregates control and treated samples into distinct groups, indicating that even a low concentration of ChNP is sufficient to induce shifts in gene expression (Kar et al. 2025). Moreover, broader and more pronounced expression changes are observed at the higher concentration of 0.3ChNP (Fig. 2).

In the 0.03ChNP group, immune-related genes were significantly upregulated, including those linked to C-type lectin receptor signaling and vesicle trafficking (see Fig. 3A C) that could act activating the immune system as previously observed by Wang et al. (2025). This immune-oriented response suggests that the lower dose of ChNP may act as an immunostimulant, potentially enhancing the shrimp’s ability to recognize and respond to environmental stressors. TRAPP complexes, which are key regulators of membrane trafficking, with molecular functions such as sulfuric ester hydrolase activity have been implicated in the modulation of immune responses through their role in vesicle transport and autophagy (Zappa et al. 2019). Thus, the enrichment of these complexes indicates a state of enhanced cellular activity. However, given that this group exhibited a slightly higher mortality rate and the presence of reddish hepatopancreas (a potential indicator of oxidative stress as reported by Abraham et al. 2013), it is possible that this immune stimulation may compromise cellular homeostasis, reflecting overactivation of stress-related mechanisms. The latter was further evidenced by the upregulation of genes such as Trs20 (a TRAPP complex component), C-type lectin-1, chitinase, hemocyanin, and cytochrome c oxidase subunit 1 (COI) (Supplementary Table S1), all of which are associated with vesicle trafficking, immune signaling, and oxidative stress responses. Excessive immune responses have been linked to tissue damage and increased mortality in shrimp under stress conditions (Xu et al. 2021). In contrast to the immune-oriented response observed in the 0.03ChNP group, the 0.3ChNP group exhibited a more regulated transcriptional response. The genes enriched in this group were associated with metabolic homeostasis, including UDP-glucose metabolism, endoplasmic reticulum (ER) stress responses, and intracellular trafficking (Fig. 4A and C). These changes suggest a cellular environment focused on metabolic balance and stress resistance, rather than activating intense defense mechanisms (Cao et al. 2024; Huang et al. 2023). This aligns with the absence of mortality and hepatopancreas coloration in this group. Upregulation of genes such as sodium/glucose cotransporter 4 and long-chain fatty acid-CoA ligase points to enhance sugar and lipid metabolism, supporting efficient energy utilization (Cao et al. 2024; Wang et al. 2023). Concurrently, the downregulation of genes involved in detoxification and proteolysis (UDP-glucuronosyltransferase, metalloendopeptidase, and senescence-associated protein) suggests a reduced need for compensatory stress responses (Supplementary Table S2) (Cao et al. 2024; Yu et al. 2024). Altogether, these patterns indicate that 0.3ChNP fosters a transcriptional environment favoring metabolic efficiency and redox balance, rather than reactive stress activation.

The observed antioxidant-related transcriptional patterns are likely attributable to the intrinsic antioxidant properties of carvacrol and thymol, polyphenolic compounds naturally present in oregano oil. These molecules are known for their antioxidant and antimicrobial effects, with total phenolic content (TPC) directly correlated to biological activity. In previous studies, oregano oil at concentrations of 0.5–10% v/v exhibited total phenolic content (TPC) values ranging from 20.97 ± 0.22 to 42.28 ± 1.15 mg GAE 100 g^− 1^, supporting its potential role in mitigating oxidative stress (Qian et al. 2022). In the current study, synthetic molecules that mimic those found in oregano oil were used at the same concentrations, demonstrating their efficacy as antioxidants. However, at higher doses, these compounds may act as prooxidants, underscoring the importance of precise dose optimization to ensure protective physiological outcomes of shrimp under thermal stress.

The qPCR results further supported these findings. The expression of antioxidant genes such as CAT, and GSH-Px exhibited a clear non-linear response, with peaks or troughs at specific doses. For instance, Mn-SOD showed a low significant regression (*R* = 0.15) despite being expressed in all dietary treatments. Mn-SOD (the primary stress defense) converts superoxide radicals to H₂O₂ which is used as a substrate for the CAT (Wang et al. 2012). So, the fact that SOD maintained a baseline without changing along treatments, reducing dependence on CAT and GSH-Px, suggests a direct ROS scavenging by the antioxidants (carvacrol and thymol). Whereas CAT and GSH-Px responded dose-dependently (Fig. 5). ChNp reduced H₂O₂ levels, implying lower oxidative stress and diminished need for CAT/GPx defense at optimal doses. CAT expression resulted in upregulation at low doses (0.03ChNP) followed by downregulation at 0.3ChNP, suggesting reduced cellular oxidative stress and a diminished need for antioxidant defense at the optimal dose, potentially indicating lower peroxide production or a shift toward redox homeostasis. When CAT expression is suppressed, cells often compensate by reducing H₂O₂ production rather than simply increasing degradation capacity. This adjustment involves improved mitochondrial function, evidenced by decreased H₂O₂ production from mitochondrial complexes I and II when catalase is overexpressed, indicating that cells can modulate upstream reactive oxygen species (ROS) production (Yao et al. 2015). In response to catalase suppression, other antioxidant systems undergo compensatory upregulation. When catalase expression is reduced, glutathione peroxidase (GPx) expression is markedly induced to maintain H₂O₂ detoxification capacity (Choi et al. 2009). Furthermore, gene expression showed similar trends to the transcriptomic results, increasing after the dose of 0.3ChNP was raised, indicating that the antioxidant activity began to act as a prooxidant at higher doses.

The transcriptomic and qPCR data together reveal a dose-dependent modulation of antioxidant and metabolic responses in *L. vannamei* under thermal stress. The 0.03ChNP treatment induced a robust transcriptional modulation of immune-related pathways and stress signaling, as evidenced by GO enrichment of receptor signaling and ER-related processes. However, at the higher dose (0.3ChNP), RNA-seq revealed a shift toward homeostatic and metabolic adjustment pathways, while qPCR showed reduced expression of antioxidant enzymes, suggesting that the optimal dose minimized oxidative stress, thereby downregulating compensatory antioxidant gene expression. This inverse correlation between antioxidant gene expression and dose suggests that 0.3ChNP may support cellular redox balance more effectively, not only by triggering antioxidant pathways, but by preventing the oxidative damage that would otherwise require their activation. The selective activation of autophagy- and vesicle-trafficking genes at low doses suggests that suboptimal delivery may trigger cellular repair and damage-containment mechanisms. In contrast, the transcriptional repression of catalase and glutathione peroxidase at the optimal dose indicates reduced oxidative burden rather than downregulated antioxidant capacity (Chen et al. 2026; Kar et al. 2025). This distinction highlights a mechanistic shift from reactive to preventive cellular strategies, in which chitosan nanocapsules at 0.3 mg/g enhance membrane stability, modulate mitochondrial signaling, and fine-tune immune pathways to maintain homeostasis under acute heat stress (Kar et al. 2025). The integration of transcriptomic and qPCR data, therefore, positions nanoencapsulated phenolic compounds as precision modulators of crustacean stress physiology, offering a novel framework for designing functional feeds that leverage targeted molecular reprogramming rather than generalized stimulation of stress responses.

The dose-dependent effects observed in this study align with previous reports (Benincá et al. 2024) on bioactive food and nanostructured additives, where low to moderate concentrations enhance antioxidant activity. In contrast, higher or imbalanced doses may lead to oxidative damage or cellular stress (Mansour et al. 2022; Ghaffarizadeh et al. 2022; Benicá et al. 2024). For example, while high dietary levels of selenium nanoparticles show initial benefits at lower doses, they can ultimately lead to oxidative stress and decreased enzyme activities, underscoring the risks associated with over-supplementation (Ghaffarizadeh et al. 2022). The transcriptomic data obtained in this study provide novel molecular evidence supporting these patterns, emphasizing that adequate dosing is crucial for achieving a favorable balance between immune stimulation and metabolic stability.

The convergence across the three analytical levels (physiological, transcriptomic, and targeted gene expression) supports the hypothesis that carvacrol and thymol nanoparticles exert a dose-dependent effect in *L. vannamei* under thermal stress. Suboptimal concentrations trigger stress responses and immune activation, whereas optimal concentrations (e.g., 0.3ChNP) promote adaptive mechanisms and metabolic balance. However, treatment with 0.8ChNP showed a slight recovery compared to 0.3ChNP, suggesting gene expression inhibition beyond the 0.3ChNP treatment, possibly as an adaptive response to oxidative stress, with modulation occurring at different doses.

This study provides critical insights for precision aquaculture nutrition, underscoring the necessity of dose standardization for functional additives. We can conclude so far that chitosan nanoparticles loaded with carvacrol/thymol at doses lower or higher than 0.3 mg per g of feed would not optimally protect shrimp against heat stress under the conditions studied. This finding highlights the importance of activating genes associated with the innate immune system and key regulators of vesicle transport and autophagy regulators. Producers must be aware of the significance of knowing the dosage present in various commercial products to ensure optimal protection. Further investigation is warranted to explore the molecular mechanisms underlying adverse responses at higher concentrations, particularly regarding vesicle trafficking and autophagy.

## Supplementary Information

Below is the link to the electronic supplementary material.

ESM 1 (CSV. 1.87KB)

ESM 2 (CSV. 8.04 KB)

## Data Availability

No datasets were generated or analysed during the current study.
